# Emergency department overcrowding: first Swiss application of the Emergency Department Work Index and risk factors for overcrowding

**DOI:** 10.3389/fpubh.2025.1691633

**Published:** 2025-11-10

**Authors:** Aline Herzog, Michael Luster, Dagmar I. Keller Lang, Ksenija Slankamenac

**Affiliations:** 1 Emergency Department, University Hospital Zurich, Zurich, Switzerland; 2 Emergency Department, Clinic Gut, St. Moritz, Switzerland

**Keywords:** emergency department, overcrowding, Emergency Department Work Index, predictors for ED overcrowding, ED staffing

## Abstract

**Introduction:**

Emergency department (ED) overcrowding is associated with increased waiting time, reduced patient satisfaction, and decreased quality of care. Numerous validated scores are available for assessing ED overcrowding. The Emergency Department Work Index (EDWIN) is the most established score for quantifying ED overcrowding. We assessed the applicability of the EDWIN in a Swiss ED and investigated further predictors of ED overcrowding.

**Methods:**

In this retrospective analysis, we included consecutive ED visits at a tertiary care hospital between 1st December and 31st December 2016. The median EDWIN per hour was defined as the first endpoint. To investigate predictors of overcrowding, we grouped ED visits with an EDWIN ≤2 as not overcrowded and those with an EDWIN >2 as overcrowded and performed multivariable regression analysis.

**Results:**

The median EDWIN score per hour was 1.4 (IQR 1.0–1.9). In 394 calculations (53%), the ED was active; 189 calculations (25.4%) showed a very busy ED; and in 161 observations (21.6%), the ED was severely overcrowded. On average, the ED was severely overcrowded six times per day. The highest EDWIN score was reported on Saturdays [mean 2.1 (SD 1.2)] and Sundays [mean 1.7 (SD 1.0)]. During weekends, overcrowding occurred from 8 p.m. to 05 a.m., and the EDWIN score ranged from 2.2 to 3.1. During the week, the mean EDWIN score ranged from 1.3 to 1.6. A reduced number of emergency physicians during night shifts (*p* < 0.001), an increased number of patients in the ED treatment area (*p* < 0.001), patients waiting for admission to the ward (*p* < 0.001), weekend periods (*p* = 0.001), and a higher number of isolated ED patients due to infections (*p* < 0.001) showed a highly significant association with overcrowding. In the case of overcrowding, the waiting time was prolonged (*p* = 0.034).

**Conclusion:**

The EDWIN score was easily applicable in a tertiary care Swiss ED, objectively displayed severe overcrowding during weekend nights, and was strongly associated with the number of available attending emergency physicians, the number of patients in the ED treatment area, patients waiting for admission to the ward, weekend periods, and the number of patient isolations.

## Introduction

In recent years, emergency department (ED) visiting rates have increased distinctly in Switzerland and around the world (1, 2). ED crowding has become a significant worldwide public health problem (3). ED overcrowding is associated with increased waiting time, prolonged ED length of stay (ED-LOS), prolonged hospital length of stay, delayed treatment, reduced patient satisfaction, and decreased quality of care, including an increase in preventable medical errors (4–16). In addition, overcrowding has also been described as a major risk factor for violence in EDs (17). The reasons for overcrowding are manifold: reduced inpatient capacity, an aging population, 24-h service availability, the lack of primary care physicians, limited ancillary service performance, a high volume of low-acuity presentations, and ED nursing staff shortages (15, 16, 18–20).

Numerous validated scores are available for assessing ED overcrowding. The Emergency Department Work Index (EDWIN) is one of the most established scores for quantifying ED overcrowding, serving as a very useful real-time warning system. It enables effective monitoring of patient flow and facilitates the timely implementation of countermeasures (21–24). The EDWIN offers an objective way to measure ED overcrowding by combining patient acuity, staffing, and bed availability into a single value. It is widely established because it is simple to calculate, can be integrated into electronic systems, and supports real-time decision-making. By identifying early signs of ED strain, it helps optimize resource allocation and improve patient flow (25). The EDWIN combines the number of patients at each triage level, the number of emergency physicians, available treatment beds, and patients waiting for in-house admission. In this way, the EDWIN represents the paradigm by dividing ED care into processes of input, throughput, and output (26).

The NEDOCS is another well-validated and widely described score (27). The NEDOCS score includes various parameters, including the use of invasive and non-invasive ventilation, which is a limiting factor in Swiss EDs. In Switzerland, patients requiring respirators are only treated in resuscitation room settings and are immediately transferred to the ICU. Consequently, patient care processes in Swiss EDs differ from those in other countries. No respiratory devices or supports are available directly in Swiss EDs; therefore, the NEDOCS score is not applicable in Swiss EDs. Van der Linden et al. modified the NEDOCS (mNEDOCS) for EDs in the Netherlands (28). Instead of using the number of ventilated patients as a variable, the mNEDOCS incorporates the number of patients in the highest triage level (i.e., critically ill patients). The organization of EDs in the Netherlands is comparable to that in Switzerland, and the mNEDOCS showed good correlations with perceived crowding. However, further studies are warranted to validate these findings and to establish the mNEDOCS more broadly. Therefore, it was not used in the present study. In addition, the hourly number of available inpatient beds is not visible or accessible in our ED; therefore, the mNEDOCS did not qualify as a standardized metric.

Another basic measure used to describe ED overcrowding is the occupancy index (OI), also known as the occupancy rate (29). The OI is defined as the ratio of the total number of inpatients in the ED to the total number of ED treatment beds during a given period. It has been shown that the simple OI is a useful instrument for describing ED overcrowding and is correlated with the “feeling of ED staff” (30). Although the OI is a simple and quickly applicable tool for assessing overcrowding in EDs, it has considerable limitations. Specifically, the OI does not address the ED processes of input, throughput, and output in depth, and it also does not take into account relevant factors such as staff resources, patient acuity, or the number and fluctuation of waiting emergency patients throughout the day (29).

To the best of our knowledge, there is no reported application of the EDWIN in a Swiss ED. To date, the EDWIN score has only been evaluated in a few European settings. Therefore, to address this gap, we evaluated ED overcrowding using the EDWIN in a Swiss tertiary care ED and investigated additional predictors of ED overcrowding. Furthermore, we also evaluated the OI as an additional, simpler instrument to describe real-time crowding in our ED.

## Methods

### Study design and general ED settings

In this retrospective analysis, we surveyed ED visits at a tertiary care hospital from 1st December to 31st December 2016. December was selected as the study month because of its comparability across years. Holidays occur at nearly the same time each year; nevertheless, emergency staffing is maintained 24/7, general practitioners operate through an on-call system, and other services remain organized in late December. In addition, the influenza season has not peaked yet. December in Zurich can be considered a climatologically average month and is typically not the coldest time of the year (unlike January or February). Extreme weather events are uncommon during this month. Other months, such as May, show greater interannual variability regarding the distributions of holidays or weather conditions.

We included all patient visits recorded at every full hour over the 31-day period without assessing any patients’ characteristics or data. All patients with mental disorders treated by the emergency psychiatrist were excluded because they did not significantly utilize human and structural ED resources.

Ethical approval or informed consent was not needed because no patient-related data were collected. We only analyzed the number of patients at each triage level, regardless of gender, age, symptoms, or diagnosis, as no further patient data were required for the calculation of the EDWIN or OI.

The annual patient volume in 2016 was 42,314, of whom approximately 8,863 patients (21%) were hospitalized. The ED was built in the 1990s (1993–1996) for approximately 22,000 patients per year and therefore has a limited number of ED beds. The number of attending physicians on duty varied: two attending physicians were on duty during day and late shifts from Monday to Friday (8 a.m. to 11 p.m.), as well as on weekends from 8 a.m. to 8 p.m. During night shifts, there was only one attending physician on duty (11 p.m. to 8 a.m.). In Switzerland, emergency medicine is not recognized as an independent board-certified specialty, but it is offered as a subspecialty qualification that can be obtained in addition to a primary specialty. Our attending physicians, with backgrounds in internal medicine, surgery, or anesthesiology—most of whom hold subspecialty certification in clinical emergency medicine—are a fixed ED team supported by residents. These include rotating residents from surgical specialties and internal medicine, as well as residents directly employed to acquire competencies in emergency medicine. For the calculation of the EDWIN score, only attending physicians are counted; residents, although actively involved in patient care under supervision, are not included in the EDWIN metric.

### Primary and secondary endpoints

The mean EDWIN score was defined as the primary endpoint to evaluate ED overcrowding. Furthermore, our secondary endpoints were to investigate the predictors of ED overcrowding and to assess the applicability of the OI compared to the EDWIN.

### The EDWIN model

The EDWIN is defined as Σn_i_t_i_/N_a_(B_T_-B_A_), where n_i_ is the number of patients in the ED in triage category i, t_i_ is the triage category, N_a_ is the number of attending physicians on duty, B_T_ is the number of treatment beds, and B_A_ is the total number of admitted patients in the ED (21).

The triage category is the Emergency Severity Index (ESI) level but in reverse order, with 5 representing the sickest patients and 1 the least acute. The ESI is a validated tool that triages ED patients into five urgency levels depending on acuity and resources: ESI 1 cases are the most urgent (life-threatening), while level 5 represents the least urgent patients. The ESI triage system has high inter-observer agreement and is associated with resource use (31).

The EDWIN was scaled as follows: from 0 to 1.5 indicates “active but manageable,” 1.5–2.0 indicates “very busy but not overcrowded,” and >2 indicates “extremely busy and severely overcrowded” (21).

### The ED occupancy index (or rate)

The occupancy index (OI, or the occupancy rate) is defined as P/B. In other words, it is the ratio of the total number of patients in the ED (P) to the total number of ED treatment beds (B) during a given period (29).

We included all patients present in the ED during each study hour, regardless of their location within the ED or treatment status. The total number of ED treatment beds was defined according to the treatment bays outlined in the ED’s original blueprint, excluding hallway locations because monitoring was not available and they are therefore not considered true ED beds (29).

An OI above 1.0 indicates that there are more patients in the ED than treatment bays. We defined this situation as “overcrowded,” consistent with other studies (14, 29). The higher the OI, the more crowded the ED.

### Data collection

We calculated the EDWIN every full hour over 24 h for 31 days, and in summary, 744 EDWIN and OI calculations were performed (21). The following data elements were extracted from the clinical information system at every full hour: number of patients in each triage level [Emergency Severity Index (ESI)], number of patients in isolation due to an infectious disease, number of patients admitted to inpatient beds, longest waiting time for admission to the ward, number of patients in the waiting room, and longest waiting time in the waiting room.

Furthermore, the ED routinely triaged patients using the ESI to assess their acuity (31).

### Statistics

Firstly, the EDWIN and the OI were calculated using the formulas defined in the previous chapter. Afterward, we grouped ED visits with an EDWIN score ≤2 as “not overcrowded” and those with a score >2 as “overcrowded,” according to the EDWIN recommendation (21).

Metric parameters were described using the mean and standard deviation if the data were normally distributed. In the case of skewed data, the parameters were reported as the median and interquartile range (IQR). Potential and assessed risk factors or predictors of ED overcrowding were investigated using a linear or logistic multivariable regression model. All results were described as point estimates with 95% confidence intervals and *p*-values (<0.05 defined as statistically significant). Statistical analysis was performed using the statistical program STATA SE (version 16, Stata Corp., College Station, TX, USA). Graphs were generated using a Python script written by ML in SciPy and Matplotlib to analyze and plot data (32).

## Results

Across December 2016, of 744 calculation points, the mean EDWIN per hour was 1.4 (IQR 1.0–1.9) (Table 1). In 394 calculations (53%), the EDWIN indicated an active ED; 189 calculations (25.4%) showed a very busy ED; and in 161 observations (21.6%), the ED was severely overcrowded (Table 1).

**Table 1:** Emergency department visits’ characteristics.

	Number of observations *N* = 744
EDWIN/hr	1.4 (1.0–1.9)
0–1.4	394 (53.0%)
1.5–2.0	189 (25.4%)
>2.0	161 (21.6%)
Occupancy index	0.7 (0.3)
<1	566 (76.1%)
=1	39 (5.2%)
>1	139 (18.7%)
Maximum waiting time for ED treatment (min)	21 (0–57)
Maximum waiting time for in-house admission (min)	45 (20–85)

All results were reported as mean (standard deviation) or median (interquartile range). EDWIN, Emergency Department Work Index; ED, Emergency Department; hr, hour.

In 566 calculations (76.1%), the OI was <1. In total, 39 calculations (5.2%) showed an OI = 1, and in 139 observations (18.7%), the OI was >1, defining overcrowding.

The median maximum waiting time for ED treatment was 21 min (IQR 0–57), and the median maximum waiting time for in-house admission was 45 min (IQR 20–85). In the case of overcrowding (EDWIN ≥ 2), the waiting time for ED treatment [median 19 min (IQR 0–55)] was not significantly different (adjusted 10.2 min, 95% CI 0.7–19.6, *p* = 0.034) from non-overcrowded situations [median 22 min (IQR 0–57)]. We observed a similar pattern for the maximum waiting time for in-house hospital admission. In the case of overcrowding (EDWIN ≥ 2), the maximum waiting time for hospital admission [median 45 min (IQR 13–75)] was not significantly different (adjusted −10.5 min, 95% CI −29.5 to 8.7, *p* = 0.29) from non-overcrowded situations [median 45 min (IQR 20–90)].

### ESI distribution during shifts

In 18.3% of all time slots during December 2016, at least one patient with triage category ESI 1 was present in the ED for treatment. In 68.1% of all time slots, at least one patient with ESI 2 was present; and in nearly all time slots (99.9%), at least one patient with ESI 3 was present in the ED for treatment. The second most frequent triage category was ESI 4; in 86.6% of all time slots, at least one patient with this lower acuity level was present in the ED for treatment. In addition, in 13.7%, at least one patient with the lowest triage category ESI 5 was present.

**Figure 1:**
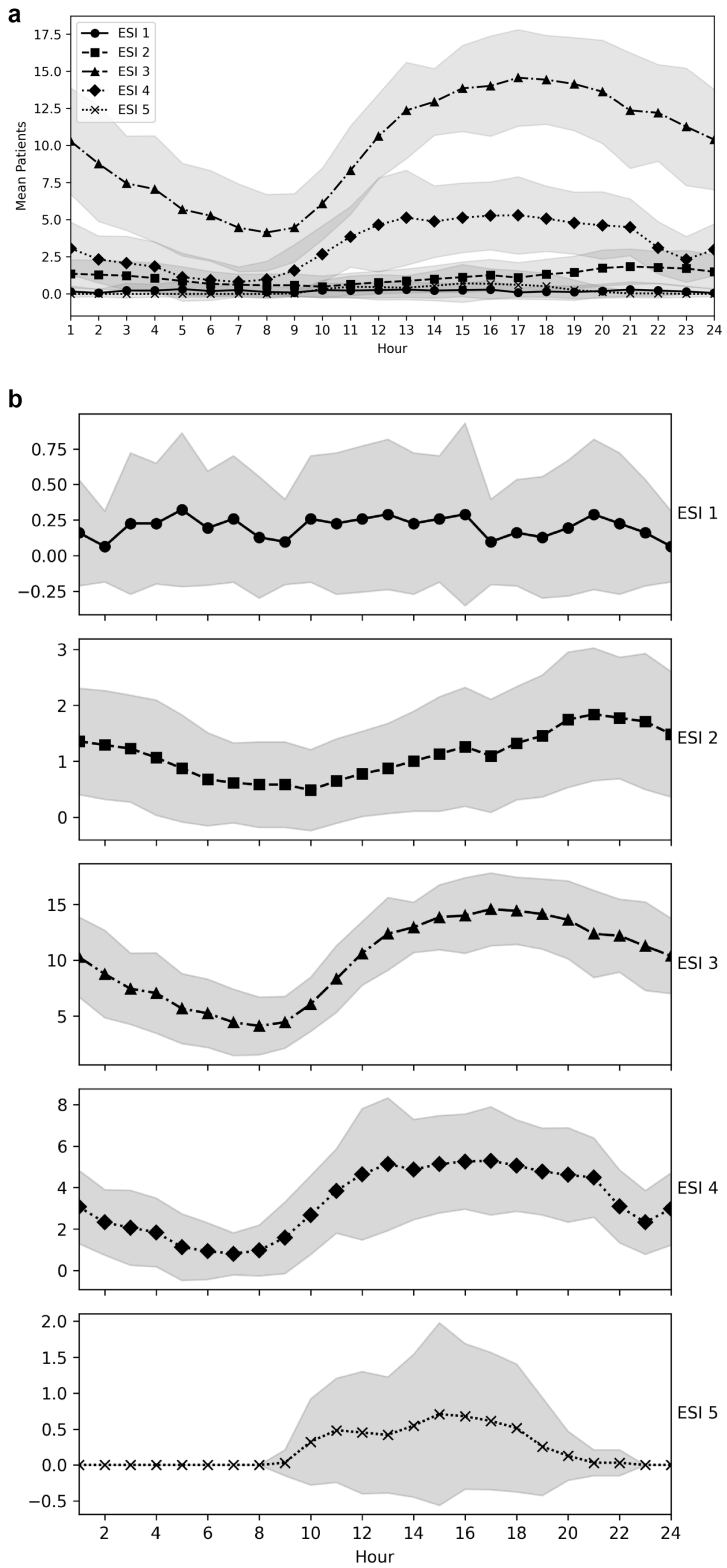
**(a)** ESI 1–5 distributions throughout the day. **(b)** ESI 1–5 distributions throughout the day, shown in separate plots for each ESI category.

Between 5 a.m. and 9 a.m., the ED had the lowest number of patients receiving treatment. During the day, there was an increase in the number of patients with triage categories ESI 3 and 4 (Figures 1a,b). From around 1 a.m., the curve flattened and the number of emergency patients decreased overall. Furthermore, there was also an increase in ESI 2 patients during the day, with a peak from 8 p.m. until midnight (Figures 1a,b).

### Overcrowding

**Figure 2:**
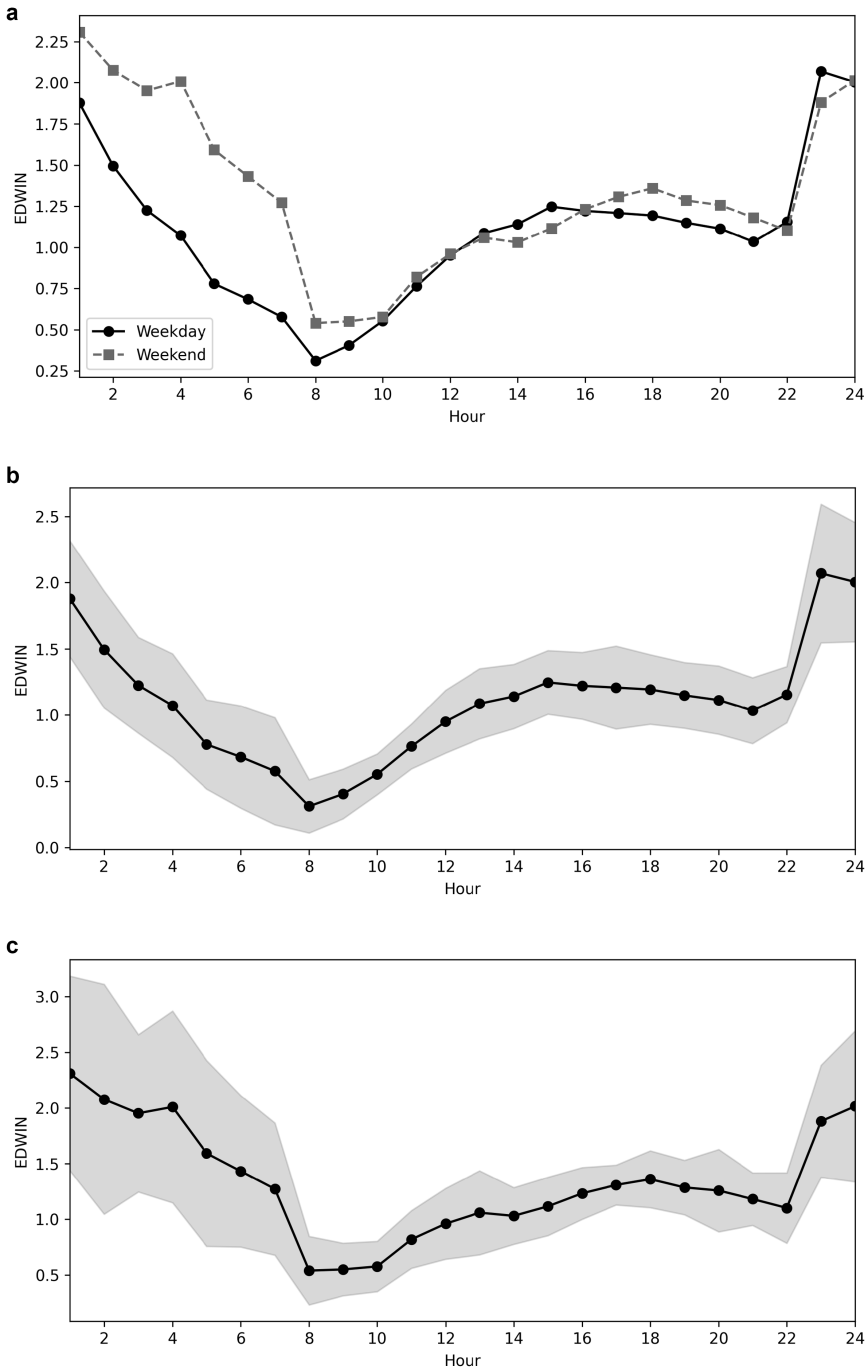
**(a)** EDWIN scores during weekdays compared to weekends, values only. **(b)** EDWIN values over time on weekdays. **(c)** EDWIN values over time on weekends.

On average, the ED was severely overcrowded six times per day (Figure 2). The lowest EDWIN scores were consistently observed in the morning. There was an increase throughout the day, with a particularly steep rise between 7 p.m. and midnight. At night, we observed a decrease in the EDWIN score (Figure 2).

The highest EDWIN score was reported on Saturdays [mean 2.1 (SD 1.2)] and Sundays [mean 1.7 (SD 1.0)]. During weekends, overcrowding (EDWIN ≥2) occurred from 8 p.m. to 5 a.m., whereby the mean EDWIN score ranged from 2.2 to 3.1 (Figures 2a–c).

During the week, overcrowding was shorter (11 p.m. to 2 a.m.), and the mean EDWIN score during the overcrowding period ranged from 2.0 to 2.7 (Figures 2a–c).

**Figure 3:**
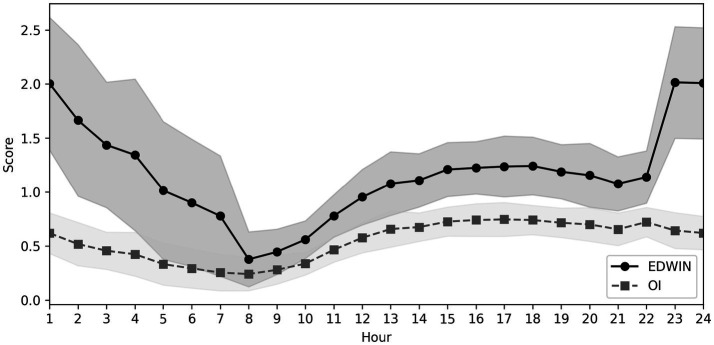
EDWIN and OI values throughout the day.

The OI showed the lowest ratios in the morning, an increase during the day, and higher ratios on weekends (Figure 3). The EDWIN and OI were strongly correlated (Spearman’s rho 0.69, *p* < 0.001).

### Predictors of overcrowding

In the multivariable linear and logistic regression analysis, a reduced number of attending emergency physicians during night shifts (adjusted difference −0.6, 95% CI −0.7 to −0.5, *p* < 0.001), an increased number of patients in the ED treatment area (adjusted difference 4.6, 95% CI 3.6–5.6, *p* < 0.001), the number of boarders waiting for admission to the ward (adjusted difference 0.5, 95% CI 0.4–0.7, *p* < 0.001), weekend periods (OR adjusted 3.5, 95% CI 2.4–5.2, *p* = 0.001), and the number of ED patients in isolation due to infections (OR adjusted 2.4, 95% CI 1.6–3.6, *p* < 0.001) showed a highly significant association with overcrowding (Table 2).

**Table 2:** Prediction of overcrowding.

	EDWIN ≤ 2 *N* = 583 (78.4%)	EDWIN >2 *N* = 161 (21.6%)	Unadjusted difference (95% CI, *p*-value)	Adjusted difference (95% CI, *p*-value)
Number of attending physicians	2 (1–2)	1 (1–1)	−0.7 (−0.8 to −0.6, *p* < 0.001)	−0.6 (−0.7 to −0.5, *p* < 0.001)
Number of patients in the ED	14 (7)	17 (5)	2.7 (1.5–3.9, *p* < 0.001)	4.6 (3.6–5.6, *p* < 0.001)
Number of patients waiting for hospital admission	0 (0–1)	1 (0–2)	0.5 (0.3–0.6, *p* < 0.001)	0.5 (0.4–0.7, *p* < 0.001)

	EDWIN ≤ 2 *N* = 583 (78.4%)	EDWIN >2 *N* = 161 (21.6%)	Unadjusted OR (95% CI, *p*-value)	Adjusted OR (95% CI, *p*-value)
Weekend (%)	162 (27.8%)	85 (52.8%)	2.9 (2.0–4.2, *p* < 0.001)	3.5 (2.4–5.2, *p* < 0.001)
Isolation (%)	315 (54.0%)	114 (70.8%)	2.1 (1.4–3.0, *p* < 0.001)	2.4 (1.6–3.6, *p* < 0.001)

CI, Confidence Interval; OR, Odds ratio, ED, Emergency Department; all results were adjusted for possible confounders such as time of day.

In the case of overcrowding, the waiting time was significantly prolonged (adjusted difference 10.2, 95% CI 0.7–19.6, *p* = 0.034).

## Discussion

The ED was severely overcrowded 21.6% of the time. During the week, this was mainly between 11 p.m. and 2 a.m., and during the weekend, this was reported from 8 p.m. to 5 a.m. The highest EDWIN score was reported on weekends. The most important factor for overcrowding was the decreased number of attending physicians during night shifts. Furthermore, a highly significant association was observed between overcrowding and the number of patients in the treatment area, the number of boarders awaiting inpatient admission, and the number of isolated patients due to infections. The first three factors are included in the EDWIN denominator and are already known risk factors. In contrast, weekend periods and isolated ED patients were identified for the first time as new predictors associated with overcrowding, as no prior studies have explicitly described these associations. These predictors reflect differences in attending emergency staffing during weekends, reduced access to referral or support services on weekends, or inefficiencies in patient-flow logistics, as well as increased capacity demands and expenses associated with patient isolation.

Compared to our data, McCarthy et al. showed a comparable but not similar pattern of crowding in the ED (29). In McCarthy et al.’s study, the median hourly EDWIN was highest between 9 p.m. and 2 a.m. and lowest at 9 a.m. In addition, the median hourly OI was highest at 6 p.m. and lowest at 7 a.m. In our study, the significant increase in crowding in the late evening is mostly due to the reduced number of attending physicians during night shifts. The differences in time frames likely reflect variations in staffing schedules between the EDs. In our ED, only one attending physician was on duty between 22:30 and 08:00, whereas McCarthy’s study involved different duty hours. Another reason for the lower crowding in our ED during the afternoon could be the opening hours and availability of general practitioners and urgent care facilities in the surrounding area.

Morley et al. used the conceptual model of Asplin et al. (26) to investigate the causes of ED overcrowding and divided the causes of ED overcrowding into three categories: Input, throughput, and output (18, 26). For example, high volumes of low-acuity presentations are described as input-related overcrowding factors (18). Based on our data, the increase in patients with ESI 3 and 4 during the day corresponds with the increase in the EDWIN score, reflecting the crowding situation in our ED throughout the day. However, the highest EDWIN scores were calculated mainly between 11 p.m. and 1 a.m., while the highest numbers of ESI 3 and 4 patients were observed in the afternoon and evening. After 9 p.m., the number of these patients decreased slowly. Instead of an increase in low-acuity presentations in the late evening, according to the literature, we reported an increase in ESI 2 patients until midnight. ESI 1 triaged patients had several peaks throughout the day, with the last peak occurring around 8 p.m., increasing and intensifying the crowding situation to the maximum during a very vulnerable phase of the day. These presentations of patients with more urgent and complex care needs (ESI 1 and 2) are also described as input-related overcrowding factors (18). The triage system of the ESI reflects urgency and complexity, at least in part. In the literature, other input-related overcrowding factors are described, such as limited access to primary care and/or diagnostic services in the community. However, in Switzerland, especially in the urban areas, we currently have a very well-developed health system without medical undersupply (33). Nonetheless, it is highly likely that this input-related overcrowding factor will also affect Switzerland and our ED in the future because of the shortage of care resulting from the decline in general practitioners. This factor needs to be observed in the future.

Furthermore, an increase in presentations by older patients was also described as an input-related overcrowding factor (18). However, previous studies have demonstrated that older patients in EDs undergo more diagnostic investigations and are more likely to be admitted to the hospital, which has been associated with an extended length of stay (34). Since we did not analyze any patient-related data, we cannot comment on an increased presentation of older ED patients.

However, the most important factor for overcrowding in our ED was the decrease in attending emergency physicians during night shifts, which represents a classic throughput limitation. Morley et al. showed that shortages in ED nursing staff are a throughput cause of ED crowding (18). Interestingly, no additional literature addressing the reduction of attending emergency physicians as a throughput factor could be found. Therefore, we are the first group to prove that a reduction in attending physicians is a significant throughput limitation leading to ED overcrowding. Other throughput factors, such as effectiveness and processing times or even delays in diagnostic tests (e.g., radiology and laboratory tests), were not assessed in this study and therefore could not be discussed.

Output causes of ED crowding include the availability of free capacities on the ward, ICU, and free telemetry places (18, 35). We did not investigate output causes in our study. Nevertheless, we assume that the association between the number of isolated patients and the overcrowding situation is due to a prolonged waiting time for in-hospital admission. It is well known, especially in our ED, that isolated beds and rooms are limited in number and ED patients become boarders due to the lack of isolation capacity in the wards. However, our study design did not allow us to demonstrate this output factor. In addition, Salehi et al. reported that patients who were older, sicker, and had telemetry requirements experienced longer boarding times (36). In addition, some studies have identified higher severity of illness or presentations with complex care needs as other causes of ED overcrowding (18, 35). We showed for the first time that isolated patients cause overcrowding in the ED due to their higher severity of illness and need for complex care. To the best of our knowledge, no other study in the literature has explicitly emphasized “isolation” as a reason for overcrowding.

Unlike the good applicability of the EDWIN in the ED, the OI could not be implemented effectively. Although there is a strong correlation between the two, the OI was unable to map the real-time situation or reflect the complex process problems such as input, throughput, and output in the ED. McCarthy et al. postulated that high EDWIN scores at these hours reflect a reduction in staffing rather than increased crowding (29). The reason for the mismatch in our ED is probably not only the change in the number of attending physicians during the night shift but also the change in the number of available beds in the ED during different shifts. In our ED, beds designated for ESI 4–5 patients were closed from 22:30 to 08:00. Patients with ESI 4–5 triage levels were managed in the central area of the ED. In addition, only one attending physician was on duty during the night shift. We agree with the theory that reduced staffing contributes to overcrowding, and moreover, we could statistically show that reductions in the number of attending physicians are significantly associated with overcrowding.

In summary, we identified two new important predictors of overcrowding: a reduction in the number of attending emergency physicians and the presence of ED patients in isolation due to an infectious disease. To minimize overcrowding, the staff schedule should be adjusted, especially in the evenings, nights, and on weekends. In 2020, we took this step in our ED and increased the number of senior medical staff to two during the night shift. Since October 2024, we have increased staffing on very busy late shifts by adding a third attending physician on duty. We have already reduced the overcrowding situation during night shifts, but the final analysis of the addition of a third attending physician needs to be performed after a pilot phase of 6 months.

Furthermore, isolated patients and other patients with complex care needs should be registered early for boarding to the wards. Junior physicians need to be trained to prepare the treatment plan early and to be aware of long boarding times.

To address overcrowding, several initiatives have been implemented since the data collection for this study—both at the regional organizational level and within our ED—which we will discuss in future studies.

## Limitations and strengths

Firstly, one limitation of the study is the focus on a single month instead of a period involving different seasons. The selection of December, a month with several holidays, could have led to an overestimation of overcrowding due to limited access to medical services (general practitioners might be closed during the Christmas holiday). However, in 2016, most of the public holidays fell on a weekend. Furthermore, in Switzerland, regional substitute services for general practitioners are well organized for weekends and holidays. Between the holidays, general practitioners are typically open. In terms of climate, December is not the coldest month of the year, and usually, there are no extreme weather conditions in the Zurich region. In Switzerland, the annual influenza season typically begins in late December or early January and peaks between mid-January and mid-February. We therefore do not assume that the month of December is a relevant source of bias. On the contrary, it is also a strength that we selected the month of December for the evaluation of occupancy, precisely because of the many public holidays and the extraordinary strain on the emergency system.

Therefore, our study also explicitly refers to the period of December 2016, before the COVID-19 pandemic, when patients had to be isolated. An analysis of the number of patients, the number of isolations, and the evolution of the EDWIN score during the COVID-19 pandemic is still pending. Our findings identify patient isolation as a key risk factor for ED overcrowding, aligning with broader observations on the systemic impact of isolation measures (37). Finally, further research will be required to determine the generalizability of these findings.

Secondly, several variables known to influence ED crowding were not included in our analysis, such as current hospital occupancy and the waiting time for laboratory or radiology results (diagnostic turnaround times), because these data were not available and therefore could not be adjusted as confounders in the analysis. In addition, no patients’ data were used in this study. Therefore, we did not analyze individual patient-level factors, such as clinical complexity, acuity, or specific case characteristics. However, the ESI triage score can provide some information about resources, acuity, and, to some extent, the complexity of a case.

Thirdly, as a tertiary hospital, we treat a highly heterogeneous and often complex patient population in close collaboration with multiple specialties. Due to legal obligations, we cannot reject admissions, except in rare cases of capacity limitations. This may have introduced a selection bias, as our cohort likely included more severely ill and multimorbid patients compared to those in non-tertiary settings.

## Conclusion

The EDWIN was successfully validated in our tertiary care Swiss ED. It objectively displays severe overcrowding during weekend nights and is strongly associated with the number of available attending emergency physicians, the number of patients in the ED treatment area, boarders waiting for admission to the ward, weekend periods, and the number of isolated patients in the ED.

To better manage ED overcrowding in the future, processes within the ED and hospital should be carried out and resources should be optimally planned. Staffing levels should be better aligned with the temporal patterns of patient flow. The most important steps include the optimization of staffing during late shifts, night shifts, and weekends to reduce periods of overcrowding and improve the quality of treatment and patient safety in the ED.

## Data Availability

The raw data supporting the conclusions of this article will be made available by the authors without undue reservation.
