# Supplementary material for: Emergency department overcrowding: first Swiss application of the Emergency Department Work Index and risk factors for overcrowding
Source: Front Public Health. 2025 Nov 10;13:1691633. doi: 10.3389/fpubh.2025.1691633 (PMC12640941; doi:10.3389/fpubh.2025.1691633)

Appendix

EDWIN values over time on Monday

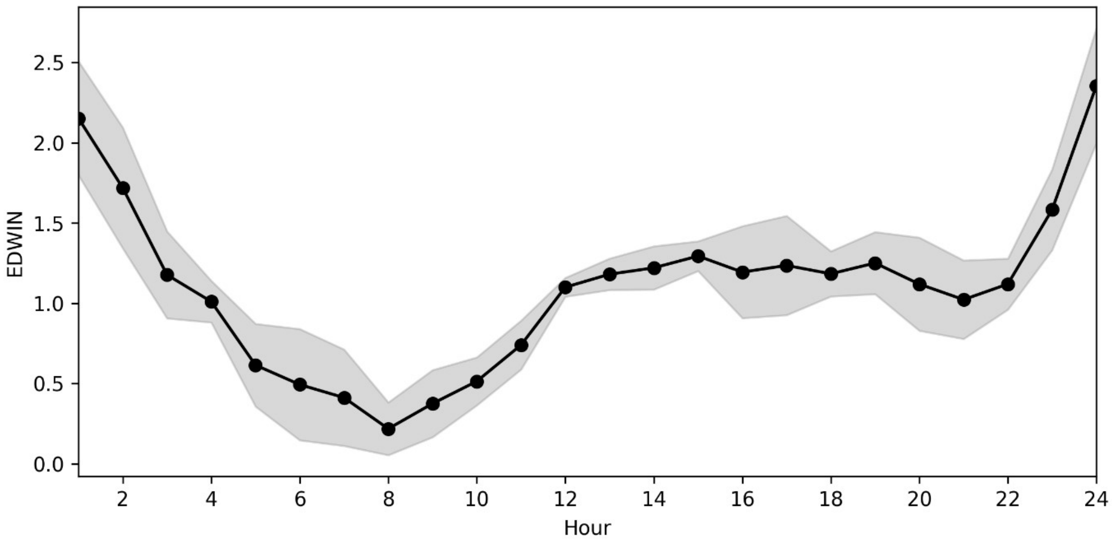

EDWIN values over time on Tuesday

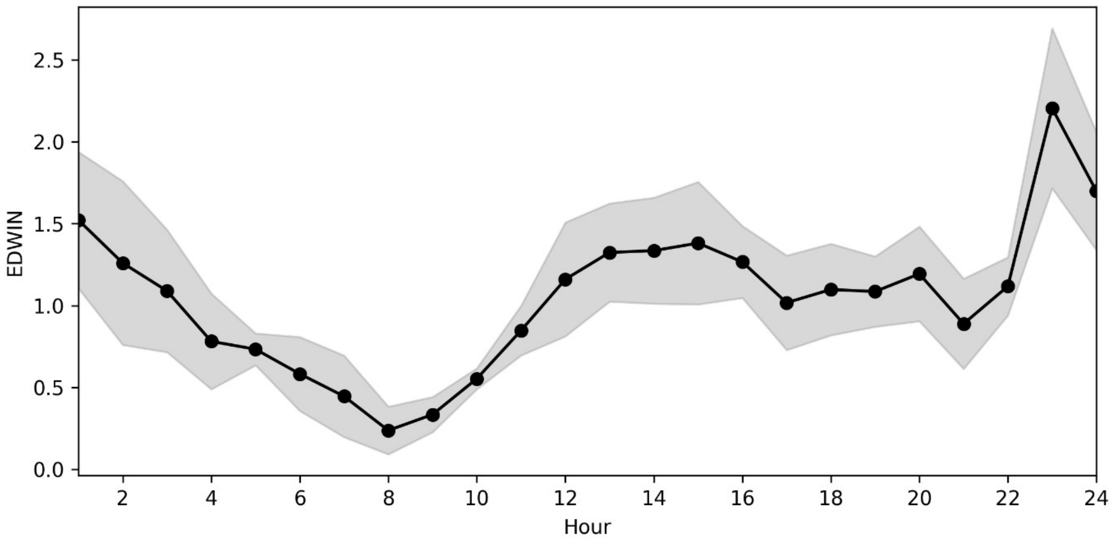

EDWIN values over time on Wednesday

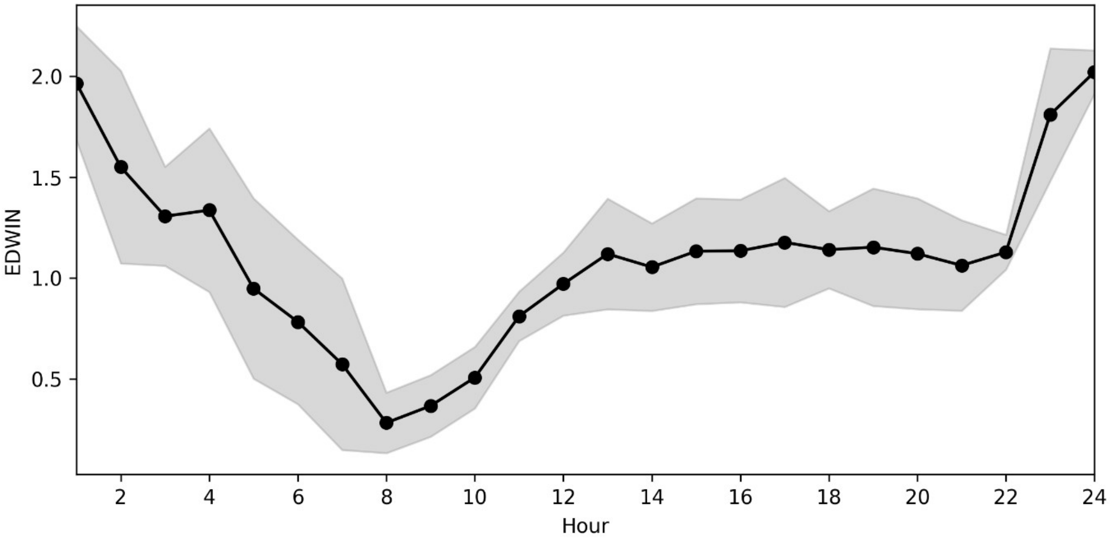

EDWIN values over time on Thursday

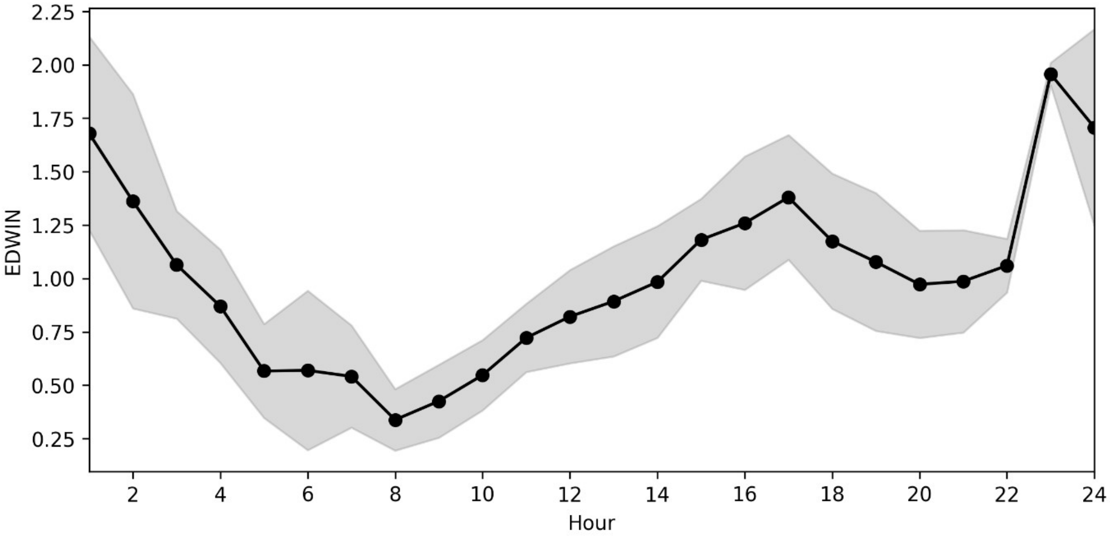

EDWIN values over time on Friday

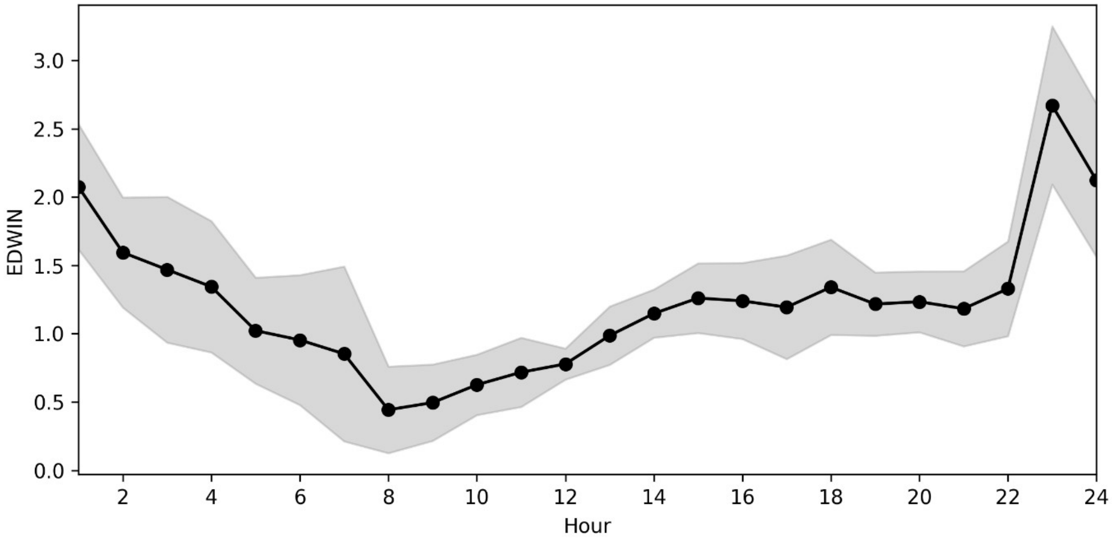

EDWIN values over time on Saturday

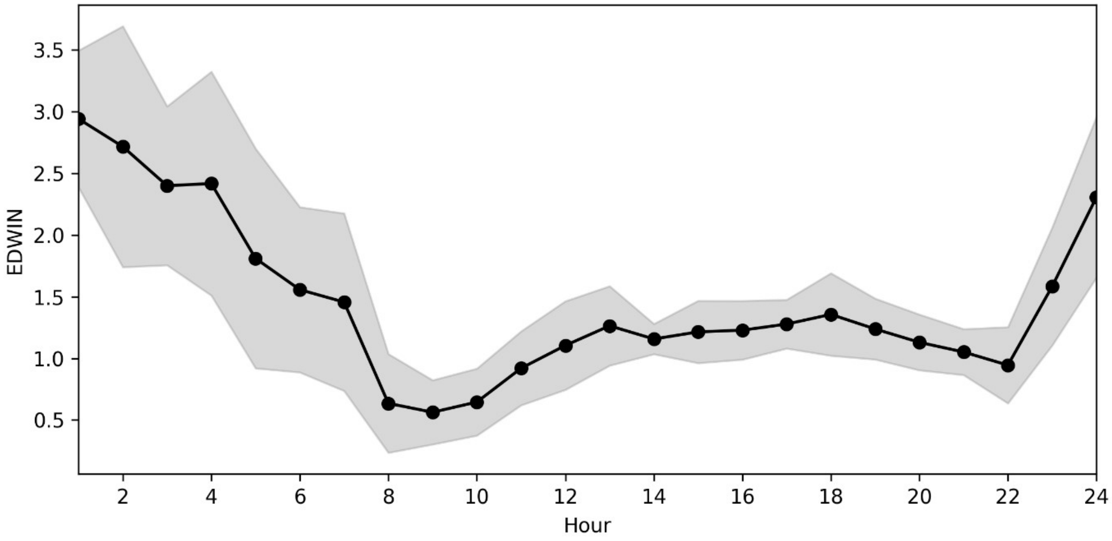

EDWIN values over time on Sunday

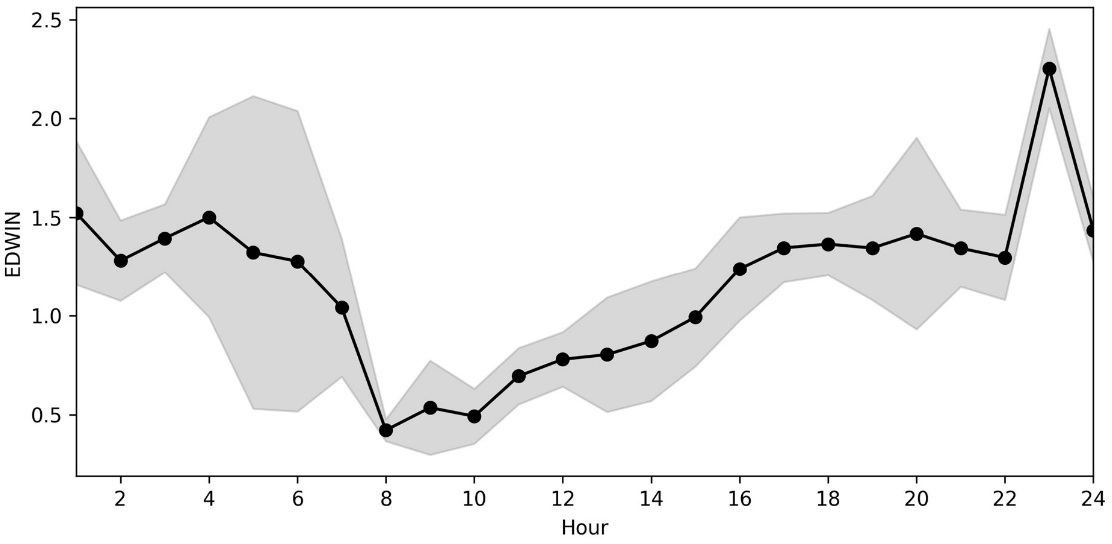

Supplement: Supplementary file 1 [file Supplementary_file_1.pdf]
